# Antibodies to hepatitis B virus surface antigen and *interleukin 12* and *interleukin 18* gene polymorphisms in hemodialysis patients

**DOI:** 10.1186/1471-2369-13-75

**Published:** 2012-08-03

**Authors:** Alicja E Grzegorzewska, Piotr M Wobszal, Adrianna Mostowska, Paweł P Jagodziński

**Affiliations:** 1 Chair and Department of Nephrology, Transplantology and Internal Diseases Poznań University of Medical Sciences, 49 Przybyszewskiego Blvd, 60-355, Poznań, Poland; 2 Department of Biochemistry and Molecular Biology, Poznań University of Medical Sciences, Poznań, Poland

**Keywords:** Antibodies to surface antigen of hepatitis B virus, Gene polymorphisms, Hemodialysis, Interleukin 12, Interleukin 18

## Abstract

**Background:**

The *interleukin (IL)18* rs360719 CC genotype is associated with the development of antibodies to hepatitis B virus surface antigen (anti-HBs) in hemodialysis (HD) patients. IL18 shares biological properties with IL12 in promoting the T-hepler 1 (Th1) system*.* We studied whether polymorphisms in the *IL12A* 3` untranslated region (UTR) and *IL12B* 3`UTR may contribute to anti-HBs development (titre ≥ 10 IU/L) in HD patients either individually or jointly with the *IL18* polymorphism.

**Methods:**

In 518 HD patients and 240 controls the *IL12A* rs568408 3’UTR G > A polymorphism was genotyped by high-resolution melting curve analysis. Polymerase chain reaction restriction fragment length polymorphism was used to detect the *IL12B* rs3212227 3’UTR A > C and *IL18* -1297 T > C rs360719 polymorphisms. The associations between the *IL12A, IL12B* and *IL18* genotypes and the risk of impaired anti-HBs development were estimated by computing the odds ratios and their 95% confidence intervals using logistic regression analysis.

**Results:**

In the logistic regression analysis, the higher frequency of rs360719 CC individually (2.9% in 207 patients without anti-HBs development vs 8.0% in 311 patients with anti-HBs development, p = 0.009) and of rs360719 CC combined with rs568408 GG (p = 0.048), rs568408 GA (p = 0.035), rs568408 GG/AA (p = 0.034) or rs3212227 AA (p = 0.046) was associated with an increased chance for the development of anti-HBs in HD patients. Patients bearing both rs568408 AA and rs360719 TT had a 10.9-fold or 8.9-fold lower chance, respectively, to develop anti-HBs compared with those carrying any other genotype (p = 0.005) or those who had both wild-type rs568408 GG and rs360719 TT (p = 0.011). Carriers of both rs3212227 CC and rs360719 TC had a 4.6-fold lower chance for anti-HBs development than carriers of any other genotype (p = 0.042).

**Conclusion:**

Development of anti-HBs in HD patients is associated with gene polymorphisms of interleukins involved in the Th1 system.

## Background

Chronic kidney disease patients on intermittent hemodialysis (HD) have been known to exhibit impaired immune system function with regards to the formation of antibodies against hepatitis B virus surface antigen (anti-HBs). Cytokines, among them interleukin (IL) 12 and IL18, play a key role in the regulation of hepatitis B virus (HBV) clearance and the immune response to HBV antigens during spontaneous natural infection [1-4] or planned vaccination [5-9].

IL-12 is a heterodimeric cytokine formed by a 35,000 dalton (Da) light chain (known as p35) and a 40,000 Da heavy chain (known as p40). The subunits p35 and p40 of IL12 are encoded by *IL12A* and *IL12B*, respectively, which are located on separate chromosomes (3p12-q13.2 and 5q31-33). IL-18 is an 18,300 Da cytokine. The human *IL-18* gene is located on chromosome 11q22.2_q22.3. IL-12 shares biological properties with IL-18, known as an interferon (IFN) -gamma inducing factor [10-13]. In mice, IL-12 p40 and IL-18 acted in concert in a poxvirus infection [14]. In other studies using *IL12 p40*−/− or *IL18*−/− mice, only IL-12 p40 or IL-18 was important for defense against human viruses adapted to the mouse [15,16]. In HD patients, the *IL18* -1297CC rs360719 genotype, attributed to increased IL-18 secretion [17], was recently connected with the development of anti-HBs [18]. It is not known whether the *IL12* genotype is concomitantly associated with the *IL18* genotype in the development of anti-HBs.

The aim of our study was to determine whether polymorphisms in *IL12A* and *IL12B* may individually or jointly with the *IL18* polymorphism contribute to anti-HBs development in HD patients. We have demonstrated that rs360719 CC individually and rs360719 CC combined with rs568408 GG, rs568408 GA, rs568408 GG/AA or rs3212227 AA are associated with an increased chance of developing anti-HBs in HD patients, whereas combined rs568408 AA and rs360719 TT or combined rs3212227 CC and rs360719 TC are associated with a lower chance of anti-HBs development. In HD the patients development of anti-HBs has been shown to be associated with gene polymorphisms of IL involved in the T-cell helper 1 (Th1) system.

## Methods

### Patients and controls

Studies were carried out in HD patients treated in 20 dialysis centers of the Wielkopolska region of Poland between February 11, 2009 and August 01, 2011. All patients with negative HBV seromarkers were vaccinated against HBV according to the standard rules for HD patients (4 vaccine doses of 40 μg each were given at 0–1–2–6 months) [19]; an anti-HBs titre was checked after 4–8 weeks from the last vaccine dose. An anti-HBs titre > 10 IU/L is assumed to be protective in vaccinated patients [20]. When an anti-HBs titre remained below 10 IU/L, vaccination against HBV was repeated. Due to fluctuations of anti-HBs in HD patients, blood testing for anti-HBs was repeated on a mandatory basis every 6 months to determine if vaccine booster doses were required.

Patients enrolled to the study had to fulfill the following criteria:

1. treatment with intermittent HD due to end-stage renal disease,

2. no signs and symptoms of acute infection with blood-borne viruses,

3. known anti-HBs titre (all available results of each patient were analyzed),

4. in patients without serological signs of HBV transmission, having an anti-HBs titre below 10 IU/L, two full vaccination series against HBV (4 doses of 40 μg each given at 0–1–2–6 months) had to be given or equivalent vaccine dosage had to be applied and the patients` anti-HBs titre had to be determined 4–8 weeks from the last vaccine dose,

5. from patients who disclosed a genetic relationship only one person could participate in the study,

6. written consent to participate in the study.

A response to HBsAg after vaccination or natural HBV transmission was considered to be positive when an anti-HBs titre exceeded 10 IU/L.

The inclusion criteria were fulfilled by 518 HD patients. These patients were divided into two groups dependent on anti-HBs development. Responders developed anti-HBs, whereas non-responders did not develop anti-HBs. Responders were the reference group for non-responders.

Group I (HBsAg non-responders, n = 207) included HD patients who did not develop an anti-HBs titre > 10 IU/L in response to HBsAg from the HBV vaccine [patients with negative total antibodies to HBV core antigen (anti-HBc), n = 177] or in response to HBsAg transmitted during natural HBV infection (patients with total anti-HBc positive, n = 30). The available medical documents for these patients did not reveal any anti-HBs > 10 IU/L.

Group II (HBsAg responders, n = 311) consisted of HD patients who developed an anti-HBs titre >10 IU/L as a result of vaccination (patients with total anti-HBc negative, n = 213) or as a result of HBV transmission (patients with total anti-HBc positive, n = 98). In some patients with a long course of renal disease, a history of vaccination effectiveness revealed periods with or without anti-HBs > 10 IU/L. If a patient had anti-HBs > 10 IU/L in the past but lost it during the course of renal disease, she/he was considered as constitutionally able to respond for HBsAg and was included into group II.

Registered blood donors from the Wielkopolska region of Poland (n = 240), qualified for blood donation according to the criteria of Polish Ministry of Health [21], served as controls for HD patients. The control persons had serum alanine aminotransferase activity not higher than 2 times the upper normal limit of the applied laboratory method. All controls showed negative blood testing for HBsAg and HBV DNA as well as for seromarkers of infection with the hepatitis C virus. Unfortunately, the vaccination rate against HBV and an anti-HBs titre were not known in these healthy individuals.

Genotype analysis for rs568408 3`UTR G > A in *IL12A*, rs3212227 3`UTR A > C in *IL12B* and -1297 C/T rs360719 in *IL18* was done in all HD patients and controls.

### Laboratory methods

HBsAg and anti-HBc were determined by Microparticle Enzyme Immunoassay (MEIA) technology (AxSYM, Abbott Laboratories, Abbott Park, USA). MEIA technology (ABBOTT, Wiesbaden, Germany) was also used for detection of anti-HBs and antibodies to hepatitis C virus (anti-HCV). HBV DNA was determined using a qualitative test COBAS AMPLICOR HBV MONITOR; HCV RNA was tested using COBAS AMPLICOR Hepatitis C Virus Test, version 2.0 (both Roche Diagnostics Ltd., Rotkreutz, Switzerland). Serum activities of liver enzymes were determined by routine laboratory methods.

### *IL12A, IL12B* and *IL18* genotyping

DNA was isolated from peripheral leukocytes using a standard salting out procedure.

The *IL12A 3’UTR* G > A (rs568408) DNA fragments were amplified using primers 5’ ATGAGGAAACTTTGATAGGATG 3’ and 5’TTCCCTTCTTAGCAATTCATTC 3’. This polymorphism was then genotyped by high-resolution melting curve analysis (HRM) using the Light Cycler *480* system *(*Roche Diagnostics, Mannheim, Germany)*.*

Identification of the *IL12B 3’UTR* A > C (rs3212227) and *IL-18* -1297 T > C (rs360719) polymorphic variants was carried out by polymerase chain reaction-restriction fragment length polymorphism (PCR-RFLP). PCR for *IL12B 3’UTR* A > C (rs3212227) was conducted using the primer pair 5’ TTAAAGACACAACGGAATAGAC 3’and 5’ TGCTTTATCAACACCATCTCC 3’. The PCR-amplified fragments of *IL12B* that were 557 bp in length were isolated and digested with the endonuclease TaqI (T/CGA) (New England Biolabs, Ipswich, USA). The *IL12B 3’UTR* A allele remained uncut whereas the *IL12B 3’UTR* C allele was cleaved into 454 bp and 103 bp fragments. PCR for *IL-18* -1297 T > C (rs360719) was conducted using the primer pair 5’ CAACAGTGATTACAAAGGAAGT 3’ and 5’ TAAATGGGTAGGAATAAGTGAGA 3’. The PCR-amplified fragments of *IL-18* 474 bp in length were digested with endonuclease NlaIII (CATG/) (New England Biolabs, Ipswich, USA). The *IL-18* C allele remained uncut, whereas the *IL-18* T allele was cleaved into 295 bp and 179 bp fragments. DNA digestion products for the *IL12B* 3'UTR A > C and *IL-18* -1297 T > C polymorphisms were separated by electrophoresis on 2% agarose gel and visualized by ethidium bromide staining. The PCR-RFLP analysis was repeated for all patient and control samples.

For quality control of the tested polymorphisms, approximately 10% of the randomly chosen samples were re-genotyped using commercial sequencing.

### Statistical methods

Differences in the distributions of demographic characteristics and selected variables between the examined groups were analyzed. The normality of distribution of variables was checked by the Shapiro-Wilk test. Descriptive statistics are presented as percentage for categorical variables, as mean with one standard deviation for normally distributed continuous variables or as median with range for not normally distributed continuous variables. The prevalence of variables was assessed by the chi square (χ^2^) test or Yates` test, as appropriate. Results were compared using Student’s *t*-test for non-paired data if distribution of variables was normal or the Mann–Whitney *U*-test for other than normal distributions.

Hardy-Weinberg equilibrium was tested by a goodness-of-fit m2 test to compare the observed genotype frequencies to the expected ones. Power analysis was conducted employing the Fisher exact test, which was available at an on-line internet service, http://biostat.mc.vanderbilt.edu/twiki/bin/view/Main/ PowerSampleSize.

The associations between the *IL12A, IL12B* and *IL18* genotypes and risk of impaired anti-HBs development were estimated by computing the odds ratios (OR) and their 95% confidence intervals (95% CI) using logistic regression analysis. To address the possibility of a gene-gene interaction effect between analyzed polymorphisms, a Multifactor Dimensionality Reduction (MDR) approach (MDR version 2.0 beta 5) was used [22].

Values of *P* < 0.05 were judged to be significant.

### Ethical issues

This study was approved by the Institutional Review Board of Poznań University of Medical Sciences, Poland.

## Results

The selected demographic, clinical and laboratory data of groups I (non-responders) and II (responders) are shown in Table 1. All patients were Caucasian. The patients in group I were significantly older and had shorter duration of renal replacement therapy (RRT). Among the 4 main causes of end-stage renal disease in groups I and II diabetic nephropathy was the most frequent with the least frequent being chronic glomerulonephritis in group I and chronic tubulointerstitial nephritis in group II. Significant differences in HBV seromarkers between groups resulted from categorization of patients to groups I or II.

**Table 1:** Data of all patients and of subjects grouped by a titre of antibodies to surface antigen of hepatitis B virus ≤ 10 UI/L (Group I) and > 10 UI/L (Group II)

**Parameter**	**All patients N = 518**	**Group I n = 207**	**Group II n = 311**	***P *value for analysis between groups I and II**
Men, n (% of all)	290 (56.0)	110 (53.1)	180 (57.9)	0.320
Age, years	62.5 ± 15.5	64.8 ± 14.9	60.9 ± 15.8	**0.004**
RRT duration, years	1.82 (0.002 – 26.1)	1.24 (0.04 – 23.8)	2.71 (0.002 – 26.1)	**0.001**
Diabetic nephropathy, n (% of all)	141 (27.2)	72 (34.8)	69 (22.2)	**0.002**
Chronic glomerulonephritis, n (% of all)	86 (16.6)	22 (10.6)	64 (20.6)	**0.003**
Hypertensive nephropathy, n (% of all)	84 (16.2)	34 (16.4)	50 (16.1)	1.000
Chronic tubulointerstitial nephritis, n (% of all)	60 (11.6)	24 (11.6)	36 (11.6)	1.000
History of acute hepatitis, n (% of all)	23 (4.4)	6 (2.9)	17 (5.5)	0.195
Positive HBsAg, n (% of all)	16 (3.1)	13 (6.3)	3 (1.0)	**0.001**
Positive HBV DNA, n (% of all HBsAg positive)	16 (100.0)*	13 (100.0)	3 (100.0)	1.000
Positive anti-HBc, n (% of all)	128 (24.7)	30 (14.5)	98 (31.5)	**< 0.0001**
Isolated positive anti-HBc, n (% of all anti-HBc positive)	17 (13.3)	17 (56.7)	0 (0.0)	**< 0.0001**
Full vaccination series against HBV with developed anti-HBs titre > 10 IU/L in anti-HBc negative patients, n (% of all anti-HBc negative patients)	213 (54.6)	0 (0.0)	213 (100.0)	**< 0.0001**
Positive anti-HCV, n (% of all)	55 (10.6)	20 (9.7)	35 (11.3)	0.663
Positive HCV RNA (n, % of all examined anti-HCV positive)	32 (58.2)	9 (45.0)	23 (65.7)	0.163
ALT (U/L)	13 (0.6 – 209)	14 (0.6 – 126)	13 (2 – 209)	0.392
AST (U/L)	14 (4 – 177)	14 (5–97)	14.5 (4 – 177)	0.570
GGT (U/L)	27 (0 – 498)	28 (5 – 308)	26 (0 – 498)	0.757

* HBeAg was checked in two HBV DNA positive patients, being on the transplant waiting list, and the results were negative. HBV viral load was determined in three patients and varied from 4,210 to 1.19E + 09 copies/mL.

Data are expressed as mean ± standard deviation or median and range.

Significant results are indicated using bold font.

Abbreviations: *ALT* – alanine aminotransferase, *anti-HBc* – antibodies to core antigen of hepatitis B virus, *anti-HBs* – antibodies to surface antigen of hepatitis B virus, *anti-HCV* – antibodies to hepatitis C virus, *AST* – aspartate aminotransferase, *GGT* – gamma-glutamyltranspeptidase, *HBeAg* – antigen e of hepatitis B virus, *HBsAg* – surface antigen of hepatitis B virus, *HBV* – hepatitis B virus, *HBV DNA* – deoxyribonucleic acid of hepatitis B virus, *HCV RNA* – ribonucleic acid of hepatitis C virus, *RRT* – renal replacement therapy.

There was no significant deviation from Hardy-Weinberg equilibrium in the genotype frequencies in HD patients of groups I and II (Table 2), and in controls (Table 3).

**Table 2:** ***IL12 *and *IL18 *polymorphisms in hemodialysis patients with a titre of antibodies to surface antigen of hepatitis B virus ≤ 10 UI/L (Group I) and > 10 UI/L (Group II)**

**Variable**	**Group I (n = 207) n (%)**	**Group II (n = 311) n (%)**	**OR (95 % CI)**	***P *value**	**Genotype frequencies (n, %) expected by Hardy-Weinberg equilibrium, Group I; Group II**
*IL12A* rs568408					
GG	157 (75.8)	220 (70.7)	1.00		152 (73.5); 222 (71.5)
GA	41 (19.8)	86 (27.7)	0.67 (0.44 – 1.02)	0.059	51 (24.4); 81 (26.1)
AA	9 (4.4)	5 (1.6)	2.52 (0.83 – 7.70)	0.094	4 (2.0); 7 (2.4)
GA/AA	50 (24.2)	91 (29.3)	0.77 (0.51 – 1.15)	0.199	*P* = 0.213; *P* = 0.783
*IL12B* rs3212227					
AA	129 (62.3)	193 (62.1)	1.00		130 (62.8;) 198 (63.6)
AC	70 (33.8)	110 (35.4)	0.95 (0.65 - 1.38)	0.796	68 (32.9); 100 (32.3)
CC	8 (3.9)	8 (2.6)	1.50 (0.55 - 4.10)	0.433	9 (4.3); 13 (1.4)
AC/CC	78 (37.7)	118 (37.4)	0.99 (0.69 - 1.42)	0.952	*P* = 0.783; *P* = 0.421
*IL18* rs360719					
TT	118 (57.0)	160 (51.4)	1.00		123 (59.4); 160 (51.4)
TC	83 (40.1)	126 (40.5)	0.89 (0.62 – 1.29)	0.544	73 (35.4); 126 (40.5)
CC	6 (2.9)	25 (8.0)	0.32 (0.13 – 0.82)	**0.009**	11 (5.4); 25 (8.0)
TC/CC	89 (43.0)	151 (48.6)	0.80 (0.56 – 1.14)	0.214	*P* = 0.330; *P* = 1.000

A significant result (a sample power 72.7%) is indicated using bold font.

**Table 3:** ***IL12 *and *IL18 *polymorphisms in hemodialysis patients with a titre of antibodies to surface antigen of hepatitis B virus ≤ 10 UI/L (Group II) and controls**

**Variable**	**Group I (n = 207) n (%)**	**Controls (n = 240) N (%)**	**OR (95 % CI)**	***P *value**	**Genotype frequencies (n, %) expected by Hardy-Weinberg equilibrium Controls**
*IL12A* rs568408					
GG	157 (75.8)	171 (71.3)	1.00		171 (71.3)
GA	41 (19.8)	63 (26.3)	0.71 (0.45 – 1.11)	0.131	63 (26.3)
AA	9 (4.4)	6 (2.5)	1.63 (0.57 – 4.71)	0.357	6 (2.5)
GA/AA	50 (24.2)	69 (28.8)	0.79 (0.52 – 1.21)	0.272	*P* = 1.000
*IL12B* rs3212227					
AA	129 (62.3)	151 (62.9)	1.00		150 (62.3)
AC	70 (33.8)	77 (32.1)	1.06 (0.71 – 1.59)	0.761	80 (33.2)
CC	8 (3.9)	12 (5.0)	0.78 (0.31 – 1.97)	0.597	11 (4.4)
AC/CC	78 (37.7)	89 (37.1)	1.03 (0.70 – 1.51)	0.896	*P* = 0.950
*IL18* rs360719					
TT	118 (57.0)	121 (50.4)	1.00		120 (50.2)
TC	83 (40.1)	98 (40.8)	0.87 (0.59 – 1.28)	0.475	99 (41.3)
CC	6 (2.9)	21 (8.8)	0.29 (0.11 – 0.75)	**0.006**	20 (8.5)
TC/CC	89 (43.0)	119 (49.6)	0.77 (0.53 – 1.11)	0.163	*P* = 0.950

A significant result (a sample power 78.1 %) is indicated using bold font.

The logistic regression analysis (Table 2), performed in HD patients with a titre of anti-HBs ≤ 10 UI/L (Group I) or > 10 UI/L (Group II), revealed that a lower frequency of the rs360719 CC genotype was individually associated with a significantly increased risk of immune non-responsiveness to HBsAg. The rs360719 CC variant was associated with a 3.13-fold increased chance to develop anti-HBs in HD patients (*P* = 0.009). The logistic regression analysis (Table 3), performed in HD patients with a titre of anti-HBs ≤ 10 UI/L (Group I) and controls, also revealed that a lower frequency of the rs360719 CC variant was associated with a significantly (*P* = 0.006) increased risk of immune non-responsiveness to HBsAg compared with the rs360719 TT variant.

Selected combined or dichotomized effects of the *IL12A* rs568408 3`UTR G > A, *IL12B* rs3212227 3’UTR A > C and -1297 T > C (rs360719) *IL-18* variants on the development of anti-HBs in HD patients are shown in Tables 4 and 5, respectively. A higher frequency of rs360719 CC combined with rs568408 GG, rs568408 GA or rs568408 GG/AA was associated in HD patients with a significantly higher chance to develop anti-HBs (*P* = 0.048, *P* = 0.035 and *P* = 0.034, respectively) compared to HD patients having both wild-type genotypes (rs568408 GG and rs360719 TT). A higher frequency of 360719 CC combined with rs3212227 AA was also associated with anti-HBs development in HD patients (*P* = 0.046) compared to HD patients bearing both rs3212227 AA and rs360719 TT. Combined rs568408 AA and rs360719 TT were associated with an 8.94-fold increased risk of non-responsiveness (anti-HBs < 10 IU/L) (*P* = 0.011) compared to the combined effects of rs568408 GG and rs360719 TT (Table 4) and with a 10.85-fold elevated risk of non-responsiveness (*P* = 0.005) compared to all other genotypes (Table 5). Combined rs3212227 CC and rs360719 TC were associated with a 4.61-fold increased risk of non-responsiveness (*P* = 0.042) compared to all other genotypes (Table 5). There were no significant effects of having the combined rs568408 and rs3212227 variants.

**Table 4:** **The selected combined effects of *IL12 *and *IL18 *polymorphisms in hemodialysis patients with a titre of antibodies to surface antigen of hepatitis B virus ≤ 10 UI/L (Group I) and > 10 UI/L (Group II)**

**Variable**	**Group I (n = 207) n (%)**	**Group II (n = 311) n (%)**	**OR (95 % CI)**	***P *value**	**Sample power (%) for significant differences**
Combined effects of rs568408 and rs360719					
rs568408 GG and rs360719 TT	90 (43.5)	115 (37.0)	1.00		
rs568408 GG and rs360719 TC	62 (30.0)	88 (28.3)	0.90 (0.59 – 1.38)	0.629	
rs568408 GG and rs360719 CC	5 (2.4)	17 (5.5)	0.38 (0.13 – 1.06)	**0.048**	45.7
rs568408 GG and rs360719 TC/CC	67 (32.4)	105 (33.8)	0.81 (0.54 – 1.23)	0.331	
rs568408 GA and rs360719 TT	21 (10.1)	44 (14.1)	0.61 (0,34 – 1.10)	0.095	
rs568408 GA and rs360719 TC	19 (9.2)	34 (10.9)	0.71 (0.38 – 1.34)	0.287	
rs568408 GA and rs360719 CC	1 (0.48)	8 (2.6)	0.16 (0.02 – 1.32)	**0.035**	45.3
rs568408 GA and rs360719 TC/CC	20 (9.7)	42 (13.5)	0.61 (0.33 – 1.11)	0.099	
rs568408 GA/AA and rs360719 TT	28 (13.5)	43 (13.8)	0.83 (0.48 – 1.45)	0.511	
rs568408 GA/AA and rs360719 TC	21 (10.1)	38 (12.2)	0.71 (0.39 - 1,29)	0.252	
rs568408 GA/AA and rs360719 CC	1 (0.48)	8 (2.6)	0.16 (0.02 – 1.32)	**0.034**	45.3
rs568408 GA/AA and rs360719 TC/CC	22 (10.6)	48 (15.4)	0.59 (0.33 – 1.04)	0.064	
rs568408 AA and rs360719 TT	7 (3.4)	1 (0.32)	8.94 (1.07 – 74.94)	**0.011**	65.5
rs568408 AA and rs360719 TC	2 (0.97)	4 (1.3)	0.64 (0.11 – 3.60)	0.602	
rs568408 AA and rs360719 CC	0 (0)	0 (0)	-	-	
Combined effects of rs3212227 and rs360719					
rs3212227 AA and rs360719 TT	76 (36.7)	102 (32.8)	1.00		
rs3212227 AA and rs360719 TC	48 (23.2)	73 (23.5)	0.88 (0.55 – 1.41)	0.602	
rs3212227 AA and rs360719 CC	5 (2.4)	18 (5.8)	0.37 (0.13 – 1.05)	**0.046**	49.4
rs3212227 AA and rs360719 TC/CC	53 (25.6)	91 (29.3)	0.78 (0.50 – 1.23)	0.283	
rs3212227 AC and rs360719 TT	40 (19.3)	52 (16.7)	1.03 (0.62 – 1.72)	0.902	
rs3212227 AC and rs360719 TC	29 (14.0)	51 (16.4)	0.76 (0.44 – 1.32)	0.328	
rs3212227 AC and rs360719 CC	1 (0.48)	7 (2.3)	0.19 (0.02 – 1.61)	0.068	
rs3212227 AC and rs360719 TC/CC	30 (14.5)	59 (19.0)	0.68 (0.40 – 1.16)	0.155	
rs3212227 AC/CC and rs360719 TT	42 (20.3)	58 (18.6)	0.97 (0.59 – 1.60)	0.910	
rs3212227 AC/CC and rs360719 TC	35 (16.9)	53 (17.0)	0.89 (0.52 – 1.49)	0.649	
rs3212227 AC/CC and rs360719 CC	1 (0.48)	7 (2.3)	0.19 (0.02 – 1.61)	0.068	
rs3212227 AC/CC and rs360719 TC/CC	36 (17.4)	60 (19.3)	0.80 (0.48 – 1.34)	0.403	
rs3212227 CC and rs360719 TT	2 (0.97)	6 (1.9)	0.45 (0.09 – 2.30)	0.307	
rs3212227 CC and rs360719 TC	6 (2.9)	2 (0.64)	4.03 (0.78 – 20.72)	0.069	
rs3212227 CC and rs360719 CC	0 (0)	0 (0)	-	-	
rs3212227 CC and rs360719 TC/CC	6 (2.9)	2 (0.64)	4.03 (0.78 – 20.72)	0.069	

Significant results are indicated using bold font.

**Table 5:** **Selected dichotomized effects of *IL12A *rs568408 and *IL18 *rs360719 in hemodialysis patients with a titre of antibodies to surface antigen of hepatitis B virus ≤ 10 UI/L (Group I) and > 10 UI/L (Group II)**

**Variable**	**Group I (n = 207) n (%)**	**Group II (n = 311) n (%)**	**OR (95 % CI)**	***P *value**	**Sample power (%) for significant differences**
Dichotomized genotypes of rs568408 and rs360719					
All other genotypes	202 (97.6)	294 (94.5)	1.00		
rs568408 GG and rs360719 CC	5 (2.4)	17 (5.5)	0.43 (0.15 – 1.18)	0.080	
All other genotypes	185 (89.4)	263 (84.6)	1.00		
rs568408 GA/AA and rs360719 TC/CC	22 (10.6)	48 (15.4)	0.65 (0.38 – 1.12)	0.112	
All other genotypes	206 (99.5)	303 (97.4)	1.00		
rs568408 GA and rs360719 CC	1 (0.48)	8 (2.6)	0.18 (0.02 – 1.49)	0.052	
rs568408 GA/AA and rs360719 CC	1 (0.48)	8 (2.6)	0.18 (0.02 – 1.49)	0.052	
All other genotypes	200 (96.6)	310 (99.7)	1.00		
rs568408 AA and rs360719 TT	7 (3.4)	1 (0.32)	10.85 (1.32 – 89.30)	**0.005**	75.2
All other genotypes	202 (97.6)	293 (94.2)	1.00		
rs3212227 AA and rs360719 CC	5 (2.4)	18 (5.8)	0.40 (0.15 – 1.10)	0.058	
All other genotypes	206 (99.5)	304 (97.7)	1.00		
rs3212227 AC and rs360719 CC	1 (0.48)	7 (2.3)	0.21 (0.03 – 1.73)	0.084	
All other genotypes	206 (99.5)	304 (97.7)	1.00		
rs3212227 AC/CC and rs360719 CC	1 (0.48)	7 (2.3)	0.21 (0.03 – 1.73)	0.084	
All other genotypes	201 (97.1)	309 (99.4)	1.00		
rs3212227 CC and rs360719 TC	6 (2.9)	2 (0.64)	4.61 (0.92 - 23.16)	**0.042**	52.6
All other genotypes	201 (97.1)	309 (99.4)	1.00		
rs3212227 CC and rs360719 TC/CC	6 (2.9)	2 (0.64)	4.61 (0.92 - 23.16)	**0.042**	52.6

Significant results are indicated using bold font.

MDR approach revealed a borderline gene-gene interaction effect between the analyzed polymorphic variants of *IL12A*, *IL12B* and *IL18* in HD patients of both groups (testing balance accuracy = 0.556, p = 0.094).

## Discussion

Vaccination against HBV resulting in the formation of an anti-HBs titre conferring protection (over 10 IU/L [20]) was reported in only 54% – 86% of HD patients using a recombinant vaccine [23-26]. HD patients that do not respond to vaccination are susceptible to HBV infection. Natural HBV transmission, if it does not lead to anti-HBs development, results in:

1. HBsAg carrier status, which is usually associated with persistent HBV replication (in this study HBV DNA was detected in all HBsAg positive patients) and infectivity to other persons,

2. occurrence of isolated anti-HBc positivity (anti-HBc positive persons are both HBsAg and anti-HBs negative), which is associated in approximately 8% of cases with HBV DNA detectable in the blood [27].

Moreover, an anti-HBs titre > 10 IU/L in HD patients is not always protective against HBV infection and seroconversions to anti-HBc positivity, also without clinical signs of disease, may occur [28].

Prevalence of HBsAg carrier status or isolated anti-HBc positivity in HD patients varies between individual HD facilities. As shown in this study, HBsAg carriers amounted for 3.1% of all HD patients and isolated anti-HBc positivity occurred in 13.3% of the anti-HBc positive HD patients. As such frequencies are in the medium range [26,29-31], these results indicate thousands of affected HD people worldwide.

The reasons of non-responsiveness to HBsAg are not fully understood. It has been shown that effective seroconversion after vaccination of HD patients depends on age, body mass, serum albumin concentration, type of dialyzer, duration of RRT, and underlying kidney disease [18,32-36]. Such risk factors of non-responsiveness as older age, shorter RRT duration and diabetic nephropathy were also present in the examined non-responders compared to responders.

Genetic aspects of responsiveness to HBsAg were also taken into account, linking responsiveness with the human leukocyte antigen system [37,38]. More recently, IL genotypes (*IL10IL-18*) were associated with anti-HBs development in response to HBsAg in HD patients [18,39].

IL12 and IL18 share biological properties through their synergism in the promotion of IFN-gamma production [11-13,40,41]. IL12, generated by macrophages, monocytes, dendritic cells, and B cells, is significantly elevated in HD patients [42-46], but despite this increase a constitutive IFN-gamma release by peripheral blood mononuclear cells (PBMCs) of HD patients may be undetectable [45]. Plasma levels of free IL18 are also increased in dialysis patients [47], but Th1 lymphocyte immunodeficiency was reported owing to the deficit of IFN-gamma [44,47]. Thereby, genes promoting expression of these IL may be helpful under specific clinical conditions. In experimental studies, mice immunized with an HBV DNA vaccine and the DNA fragments containing the p35 and p40 coding sequences of murine IL-12 demonstrated increased production of both immunoglobulin (Ig) M and IgG anti-HBs titers [5]. Mice vaccinated with a recombinant plasmid carrying the gene encoding HBsAg linked to a DNA segment encoding full-length murine IL18 revealed significant serum anti-HBs IgG response after two intramuscular injections [8]. These effects may be related to the indirect influence of these cytokines on anti-HBs development, may be mediated through the observed increased INF-gamma production or both. In this study neither the *IL12A* rs568408 nor the *IL12B* rs3212227 polymorphic variants were individually associated with anti-HBs development in the examined HD patients as was shown for *IL18* rs360719 CC. However, patients bearing the *IL18* rs360719 CC genotype had a greater chance to develop anti-HBs also when occurring concomitantly with the *IL12A* rs568408 GG, *IL12A* rs568408 GA or rs3212227 AA polymorphic variants, but the *IL12A* rs568408 AA and *IL12B* rs3212227 CC variants occurring together with other than the CC variant of *IL18* rs360719 were negatively associated with anti-HBs development.

Liu et al.. [48] using http://pupasuite.bioinfo.cipf.es/http://exon.cshl.edu/ESE/ and http://genes.mit.edu/burgelab/rescueese found that rs568408 may disrupt exonic splicing enhancers. They hypothesized that IL12 mRNA may be unstable or that IL12 secretion may be lower due to disrupted exonic splicing, which was suggested as a functional characterization of *IL12A* rs568408. Decreased IL12 secretion results in lower INF-gamma levels [41]. Liu et al. [48] showed that the *IL12A* rs568408 AA and *IL12A* rs568408 GA/AA genotypes were more frequent in patients suffering from hepatocellular carcinoma compared to controls. These patients were HBsAg positive in 73.5% of cases, thereby, near exclusively anti-HBs negative, whereas controls were HBsAg positive in 12.6% of cases. Although the anti-HBs titre is not mentioned in the study by Liu et al. [48], their data directly indicate that A allele of *IL12A* is associated with a lack of anti-HBs development. In our study, HD patients with an anti-HBs titre ≤ 10 IU/L had a higher frequency of *IL12A* rs568408 AA in association with *IL18* rs360719 TT than did HD patients with anti-HBs titre > 10 IU/L. Studies by Sánchez et al. [17] indicate that inhibitory transcription factor OCT-1 binds to the T allele but not to the C allele at position −1297 (rs360719). The rs360719 T allele was identified as a possible major repressor site in the *IL-18* promoter. This suppression would result in reduced IL-18 production.

Functional characterization of *IL12B* rs3212227 3’UTR A/C is not clear. Morahan et al. [49] observed that the rs3212227AA genotype was associated with a significantly elevated expression of IL12 in Epstein-Barr virus transformed human cell lines. Similar results were reported using peripheral lymphocytes: the expression of the 1159A allele was approximately 50% higher than that of the 1159 C allele [50]. On the other hand, Seegers et al. [51] correlated a *Taq*I polymorphism (C/C) in *IL-12B p40* 3`UTR with increased IL-12B p70 secretion by stimulated monocytes. Additionally, Yilmaz et al. [52] associated the 1188A/C polymorphism in the 3`UTR of the *IL-12B* gene with the expression of IL-12B mRNA and IL-12B secretion level from lipopolysaccharide (LPS) and purified protein derivative (PPD) stimulated PBMCs. Individuals +16974CC homozygous at the *IL12B* 3'UTR had significantly higher IL-12 secretion levels from LPS and PPD stimulated PBMCs than AC heterozygotes or AA homozygotes [52]. Sánchez et al. [17] found a significant increase in the relative expression of IL-18 mRNA in individuals carrying the rs360719 C allele. As shown in this study, combined effects of *IL-18* rs360719 CC and *IL12B* rs3212227 AA were positively associated with anti-HBs development. In this case, an elevated expression of IL18 could be accompanied by increased expression of IL12B. Thereby, our results confirm previous results indicating that *IL12B* rs3212227 AA is associated with elevated IL12 levels [49,50].

It has been discussed that genetic investigations could help in the development of new and improved vaccines against HBV and may eventually reduce the proportion of vaccine failures [53]. It has been shown that the use of exogenous IL12 as an adjuvant to augment anti-HBs development in response to vaccines against HBV [54,55] may help overcome at least some immunologic deficits of genetic origin. There are also experimental studies that take advantage of the recombinant plasmid carrying gene encoding the HBsAg linked to DNA segment encoding full-length murine IL18 [8]. We have suggested such a vaccine for non-responders bearing other *IL18* polymorphic variants than −1297 CC rs360719 [18]. However, at present we are very careful in our conclusions, because associations that have been found between polymorphic variants of genes encoding cytokines may disturb the unique homeostasis between cytokines with opposing action. Thus, practical significance of the obtained results cannot yet be declared, although it does indicate a necessity and implications for further studies.

There are some limitations of our study which need to be addressed. The measurement of IL12A, Il12B, IL18 and INF-gamma serum concentrations, especially during vaccination or natural HBV transmission, was not possible due to a lack of patient material, although it could provide further information on mechanisms of anti-HBs formation in relation to the respective genotypes. An other limitation of our study is the moderate number of the examined patients, especially since genetic influences on responsiveness to HBsAg with anti-HBs development were shown in homozygotes carrying polymorphic variants of low frequency, which limits the statistical power of the study. Numerous analyses showed borderline significance and were not used to support our conclusion, as they may indicate the involvement of ILs of the Th1 pathway in the immune response to HBsAg. Therefore, large population-based studies are warranted to further elucidate the impact of the examined IL polymorphisms on anti-HBs development. Finally, we would like to stress that our results were obtained in Caucasian HD patients living in the Wielkopolska region of Poland. Prevalence of rare homozygotes of both *IL12* and *IL18* may vary in other ethnicities. The frequency of the *IL12A* rs568408 AA polymorphism in a Chinese control population was 1.2%, and 18.7% for *IL12B* rs3212227 CC [48], whereas in our Caucasian controls the respective frequencies were 2.5% and 5.0%. Prevalence of *IL18* rs360719 CC was 5.7%, 5.5% and 6.1% in Spain, Italy and Argentina, respectively [17]. In controls from the South Moravia region (more proximal to Poland), the *IL18* rs360719 CC frequency was 8.0% [56]; in our study this frequency was 8.8%. The ethnic differences in IL genotype prevalence may modulate the effect of ILs on the humoral and cellular immune response, but further investigations are needed for *IL12* and *IL18*.

## Conclusions

1. Polymorphisms in *IL12A* and *IL12B* may jointly with *IL18* polymorphism contribute to anti-HBs development in HD patients.

2. In HD patients, the development of anti-HBs is associated with gene polymorphisms of ILs involved in the Th1 system.

## Competing interests

The authors declare that they have no competing interests.

## Authors’ contributions

AEG gave a conception, participated in the design of the study, performed a clinical interpretation of the data and wrote the manuscript. PMW performed the statistical analysis and participated in its interpretation. AM performed MDR analysis and interpreted its results. PPJ carried out the molecular genetic studies and participated in the study design. All authors read and approved the final manuscript.

## Pre-publication history

The pre-publication history for this paper can be accessed here:

http://www.biomedcentral.com/1471-2369/13/75/prepub
